# Innovative method of alopecia treatment by autologous adipose-derived SVF

**DOI:** 10.1186/s13287-021-02557-6

**Published:** 2021-08-28

**Authors:** Sun Jong Kim, Myung Jin Kim, Young Jun Lee, Joo Chan Lee, Ji Hyang Kim, Do Ha Kim, Young Hoo Do, Jun Woo Choi, Sung Ill Chung, Byung-Rok Do

**Affiliations:** 1 Department of Bioconvergence, HurimBioCell Inc., Seoul, Korea; 2 Biotechnology Research Institute, Hurim BioCell Inc., Seoul, Korea; 3 Top Plastic Surgery, Teheran-ro 111, Gangnam-gu, Seoul, Korea; 4 Department of Applied Statistics, College of Natural Science, Hanyang University, Seoul, Korea

**Keywords:** Stromal vascular fraction (SVF), Alopecia, Hair loss, Baldness

## Abstract

**Background:**

Alopecia refers to a condition developed by gradual reduction of hair loss by various abnormal causes such as endocrine system, genetic factors, and stress. Stromal vascular fraction (SVF) isolated from the fat is one of the latest innovative solutions in the field of regeneration therapy. We focused on presenting effectiveness of clinical cases to improve AGA through transplantation of autologous SVF into the scalp.

**Objective:**

To confirm the efficacy of the autologous SVF usage to the patients with AGA.

**Methods:**

Nine patients (age range 43–64 years; 4 men, grade IV to V and 5 women, grade I to III), who are suffering from androgenic alopecia (AGA), were treated with single transplantation of autologous SVF in the upper scalp. Autologous SVF was isolated and characterized prior to the injection of live 7–9 × 10^6^ cells into the patients’ treatment site. The hair loss improvement effect was assessed by three test criteria: hair skin quality, hair thickness and hair density 3 and 6 months after post-injection compared to pre-injection status.

**Results:**

Hair density of SVF-treated side was significantly increased after 3 and 6 months of transplantation compared to non-treated side (*P* = 0.01 and *P* = 0.009 per each). And significant improvement in the score of the keratin on the scalp was seen in the injected area as compared to the non-injected area 6 months after transplantation (*P* = 0.032). Although thickness increase was observed at 3 and 6 months after transplantation, there was no statistical significance (*P* = 0.142 and 0.155, respectively).

**Conclusions:**

One transplantation of autologous SVF for the AGA patients, hair density and score for the keratin were significantly increased within 6 months. This study shows that SVF is a very effective way to treat hair loss and most of subjects are satisfied with the result after treatment.

## Introduction

Androgenic alopecia (AGA) means the lack of body hair, especially follicle of hair, due to various reasons such as endocrine abnormalities, genetic factors, stress, sex and age [1, 2]. It is defined as baldness when hair follicle cells are completely destroyed, and it is not likely that the hair grows back, and it can be distinguished clearly from a normal person by withdrawal of the frontal hair line. In case of AGA, there is no special solution with present medical technology and although finasteride and minoxidil have been approved by FDA, it merely delays the progress of AGA and a fundamental treatment has not yet been reported [3–5].

In general, AGA treatments can be divided into surgical and non-surgical methods. A representative surgical treatment is to transplant hair from the occipital to the hair loss area which does not make the hair thin or removed [6–8]. In case of non-surgical treatments, there is no proven method objectively and in order to treat AGA, it is known to take a 5-red reductase inhibitor that suppresses production of DHT hormone and if taking the medicine is stopped, it is estimated that alopecia occurred again by reproduction of DHT hormone. AGA is known as a disease in which one's immune cells generate immune inflammatory response to hair roots and become to lose hair in the end. Although various treatments have been known to date, it is not known there is any effective method to inhibit the progression of AGA by stimulating the scalp itself or to regulate the cycle of hair which can restore AGA [9, 10].

According to some reports on the efficiency of AGA improvement using biological formulations such as culture fluid of platelet-rich plasma (PRP) or adult stem cells, the results of the treatment are known to be insignificant. According to a recently published academic paper, the result of clinical treatment using SVF by liposuction in patients’ abdomen and thigh has been statistically effective. Treatments applied in the area of AGA using adipose-derived stem cells or its culture fluid have been continuously reported [11].

Among various clinical studies on mesenchymal stem cells (MSCs), the treatments using SVF from the adipose tissues are reported to be effective on degenerative arthritis, treating wound and damaged tissue regeneration. SVF has been identified through several studies for the effectiveness of neovascularization stimulation and inflammatory change reduction, and the stability has been reported with several clinical cases [12–14]. SVF includes not only stem cells but also vascular and immune cells, and is known to restore damaged body parts, activating surrounding tissues by secreting various cytokines depending on the environment [15]. The hair cycle of scalp is also known to be affected by various environment factors, and the vascularity on the scalp may also be an important factor in the health of the hair root [6–8]. So, it is estimated that improvement of the condition on the scalp SVF with plenty of stem and vascular cells contributes to restoring hair cycle. In this study, it is aimed to verify the improvement of alopecia by using SVF separated from autologous adipose-derived tissues [16–19]. This report is a prospective preliminary study which introduces SVF in the treatment of AGA and regarded to be highly utilized in clinical cases which can suggest a new standard to AGA treatment.

## Materials and methods

### Subjects

The medical records of patients were reviewed to collect patients who treated with autologous SVF for AGA at the plastic surgery clinic (TOP Plastic Surgery, Seoul, Korea). The medical status and hair loss history were analyzed through the questionnaire, and a physical experiment was conducted to diagnose AGA grade. Healthy adult men and women patients between 43 and 64 were enrolled in this study, and androgenic baldness rated recorded using the Norwood–Hamilton grades and Ludwig scale. Over a period of 6 months, nine patients (4 male and 5 female) were followed the results. Table 1 describes the enroll criteria for subjects. All subjects had a normal body mass index (BMI) range, generally healthy, and had no history of underlying disease. Written consent for the portrait rights and publication was taken from all patients, and IRB approved the concerned clinical trial process. Table 2 summarizes patients’ information, including the age, gender, volume of adipose tissues, isolated SVF and injected SVF cell counts.

**Table 1:** Subject recruitment criteria in the study

Inclusion	Men or women with AGA symptoms
	Healthy subjects aged 43–64 years who provide written consent suitable to this study
	Subjects with grades II–VI Norwood–Hamilton or Class I–III Ludwig
	Subjects with severe hair loss within past 12 months
	Subjects who do not have any specific diseases or abnormal health conditions in the interview in relation to AGA
	Subjects with required proper hypodermic fat from the abdominal or thigh without any problem in the process of liposuction
	Women who are not pregnant during the test period
Exclusion	Subjects with prescription for inflammation, infection, malignancy, allergic diseases, autoimmune diseases, pregnancy, diabetes, anti-thrombotic drugs, etc.
	Subjects who do not experience any improvement previously in the hair loss treatments, or who have sensitive skin and suffer from the skin irritation and scratch on the surface of scalp during treatments
	Subjects with psychiatric history or other physical illness within 30 days of this clinical trials
	Subjects with a history of abnormalities in the blood vessels, heart, lungs, kidneys, digestive organs, liver, central nervous systems, etc., or who may have a risk of developing these diseases
	Women who are during pregnancy or plan on being pregnant throughout the study

**Table 2:** Patient profiles and transplanted SVF information

Subject’s No	Age	Sex	Stage	Aspirated adipose tissue/cc	Harvested SVF volume/cc	Live SVF cells × 10^6^/cc	Injected live cells × 10^6^/48 spot
1	51	M	Class IV	70.2	15.4	1.62	9.55
2	51		Class IV	90	17.0	1.62	7.68
3	54		Class V	80	17.0	0.65	7.68
4	56		Class V	90	17.0	0.69	7.68
5	43	F	Class II	90	14.6	1.01	7.68
6	44		Class II	90	17.0	1.22	7.68
7	48		Class I	90	15.3	1.29	7.68
8	59		Class II	80	17.0	1.32	7,68
9	64		Class III	90	16.0	2.15	7.68

The viability of SVF cells was 95.4 ± 3.6% (range 91.8–99%) before and after needle injection

### Liposuction, harvesting, and preparation of adipose tissue processing

On the day of surgery, we firstly checked the subjects’ medical conditions regarding lipoaspiration and treatment of SVF. After local anesthesia, tumescent fluid was infiltrated, and adipose tissue isolated with a 60-mL clogged syringe from the abdominal subcutaneous layer by using a cannula with a diameter 3.0 mm (Medical land, Seoul, Korea). An average 90 cc of adipose tissue was extracted from the patients and split into two 50 ml of syringes.

HuriCell System (HC1500, HurimBioCell, Seoul, Korea) was used to obtain SVF according to the manufacturer’s instructions. In general, tissue processing within the HuriCell device uses a sterile disposable set and the collagenase Type I (Sigma-Aldrich Corp., Seoul. Korea). Once the HuriCell disposable kit is placed within the device, the system performs an auto-check to ensure that it is perfectly sealed. Adipose tissues then move into the processing canister and are washed by warmed saline to decontaminate waste and mixed blood. HuriCell device calculates the total amount of enzyme based on the volume of tissue. The adipose tissues are continuously agitated in the process of enzymatic digestion for 30 min at 37 °C. Once digestion is complete, the SVF fraction is moved to another chamber, washed further 3 times and centrifuged. After the process is completed, SVF is resuspended in 15–17 cc of saline and transferred with a syringe. The isolation of SVF took 75–90 min depending on the amount of adipose tissue. We filtered SVF using 70-μm cell strainer (BD Biosciences, Inc., San Jose, CA, USA) to remove the aggregated cells. The viability of SVF recovered from tissue in each patient was determined by a semi-automated ADAM MC Cell Counter (NanoEnTek, Seoul, Korea).

### Phenotyping SVF

Expression of surface markers on SVF was determined by Attune™ NxT Flow Cytometer (Thermo Fischer Scientific, USA). Using each specific anti-human antibody (BD Pharmingen, USA) CD31, CD34, CD45, CD73, CD90, and CD105 SVF surface marker were used for flow cytometric analysis. Isotype control staining was performed with IgG1-FITC and IgG2b-PE. Data represent the percentage of positive cells for each marker analyzed on SVF and are means ± SD.

### Treatment of SVF

Without local anesthesia, we disinfect with chlorhexidine on the upper front, biparietal and pyramidal area. Using a syringe 3 cc (30 gauge), SVF is transplanted into the scalp at 4 mm depth. In advance, 2 cm in a square area (4 sites) to be transplant SVF is marked in the scalp and then, 0.15 cc per spot, 48 spots—total 7.2 cc of SVF is perpendicularly injected. Without removing immediately upon after injecting, we stuck the syringe in the scalp for approx. 2 s to prevent leakage of the injected cells from the transplant site. After treatment, all subjects were prescribed antibiotics for 3 days and recommended not to wash their hair on the day of SVF injection and not to do excessive exercise for approx. 1 week. On the day of the treatment, all subjects can enjoy their daily lives.

### Measurements and statistical analysis

We randomized the treated side of the patients, and the improvement was assessed by two independent observers using Aroma Smart Wizard system (ASW200, Aram Huvis, Seoul, Korea) without physicians before and after SVF treatment. After 1, 3 and 6 months, the patients’ AGA improvement status was confirmed by a physician and AGA standard scores with photographs were taken by two blinded observers at the same distance each time. Each side for observation symmetrically divided in half based on 4 cm from the hairline. Hair density (per cm^2^), hair thickness, scalp status, keratin of scalp, scalp sensitivity, scalp sebum, hair pore status, and cuticle status were analyzed automatically by average value measured over at 3 random sites using ASW200.

To avoid disturbance by medications administered to the patient, all values in these statistics used the difference of a median value between treated and the non-treated sides. Statistical significance was used by the Wilcoxon signed-rank test, a nonparametric statistical method corresponding to the student's *t* test. It was statistically considered significant if the *P* values are less than 0.05.

## Results

Nine patients recruited from November 2020 to May 2021 were divided into two parties according to gender: male (*n* = 4) and female (*n* = 5). We randomly selected the patient’s treatment side. We managed the isolation and transplantation of SVF not exceeding 120 min. In accordance with AGA medical prescription guidelines, we treated 1 mg of finasteride, 0.5 mg of dutasteride for men and 3% minoxidil foam for women.

Table 3 shows the results of the basic phenotype for the isolated SVF. The transplanted cells which were not purified showed a clearly heterogeneous population expressing not only ADSC markers but also the hematopoietic, immune-related cells and endothelial along with specific high levels of CD34 [19]. Compared with the conventional standard manual purification with HuriCell, typical SVF characteristics, the viability, and the doubling time were not different significantly (data not shown). The greatest advantage of isolating SVF by machine is the reproducibility of the procedure, thereby reducing patient-to-patient variability in the isolated cell population, which is an important parameter to control when treating with stem cells in the clinic (Tables 4, 5).

**Table 3:** Phenotyping of cell surface markers on SVF

Marker	Percentage of gated (Means ± SD, *n* = 3)	Characterization
CD31	33.88 ± 11.45	Endothelial
CD34	55.65 ± 11.85	Hematopoietic
CD45	2.33 ± 2.06	Immunological
CD73	12.53 ± 13.39	Mesenchymal
CD90	58.52 ± 11.19	Mesenchymal
CD105	10.03 ± 8.44	Mesenchymal

All subjects (*n* = 3) performed in duplicate experiment, and the number is mean ± SEM. The characteristics of isolated cell using HuriCell device show similar patterns as published data

**Table 4:** The change of hair density before and at 1, 3 and 6 months after SVF transplantation

Patient’s no.	Age	Sex	Hair density before treatment (hair/cm^2^)		Hair density 1 month (hair/cm^2^)		Hair density 3 months (hair/cm^2^)		Hair density 6 months (hair/cm^2^)	
			Non-treated	Treat	Non-treated	Treat	Non-treated	Treat	Non-treated	Treat
1	51	M	40	50	40	50	55	75	65	90
2	51		30	40	45	50	50	65	65	80
3	54		25	30	40	55	45	75	55	90
4	56		50	45	60	50	60	55	70	85
5	43	F	40	45	40	45	45	70	60	100
6	44		60	55	70	65	75	85	85	95
7	48		55	55	55	60	60	75	70	95
8	59		30	30	35	45	45	65	75	80
9	64		70	40	70	45	55	85	75	95

Average value of hairs was measured automatically over at 3 random sites using Aroma Smart Wizard system (ASW200)

**Table 5:** The change of hair diameter before and at 1, 3 and 6 months after SVF transplantation

Patient’s no.	Age	Sex	Hair thickness before treatment (mm)		Hair thickness 1 month (mm)		Hair thickness 3 months (mm)		Hair thickness 6 months (mm)	
			Non-treated	Treat	Non-treated	Treat	Non-treated	Treat	Non-treated	Treat
1	51	M	0.022	0.028	0.028	0.031	0.044	0.042	0.047	0.055
2	51		0.028	0.023	0.03	0.023	0.033	0.029	0.047	0.045
3	54		0.018	0.021	0.028	0.029	0.032	0.031	0.049	0.051
4	56		0.029	0.027	0.03	0.033	0.022	0.044	0.033	0.047
5	43	F	0.034	0.028	0.03	0.031	0.04	0.046	0.06	0.063
6	44		0.033	0.035	0.037	0.036	0.038	0.038	0.053	0.056
7	48		0.034	0.025	0.036	0.038	0.048	0.052	0.066	0.082
8	59		0.029	0.038	0.038	0.046	0.04	0.048	0.062	0.063
9	64		0.06	0.038	0.06	0.04	0.061	0.065	0.063	0.068

Average value of the hair diameter was measured automatically over at 3 random sites using Aroma Smart Wizard system (ASW200)

Patients in each group underwent transplantation for the same quantity of total live SVF (except 1 patient) according to individual hair loss type. The mean age of patients was 53 ± 1.22 in male group and 51.5 ± 3.43 in female group. A total of 3 patients (30%) had an AGA family history, and the other 6 patients (70%) were experiencing serious hair loss in the recent past year. One female (20%) showed Ludwig scale type III, and three females (60%) did Ludwig scale II. In case of male patients, most of them showed Hamilton–Norwood scale type IV or V. Any side effects were not observed in all subjects.

**Fig. 1:**
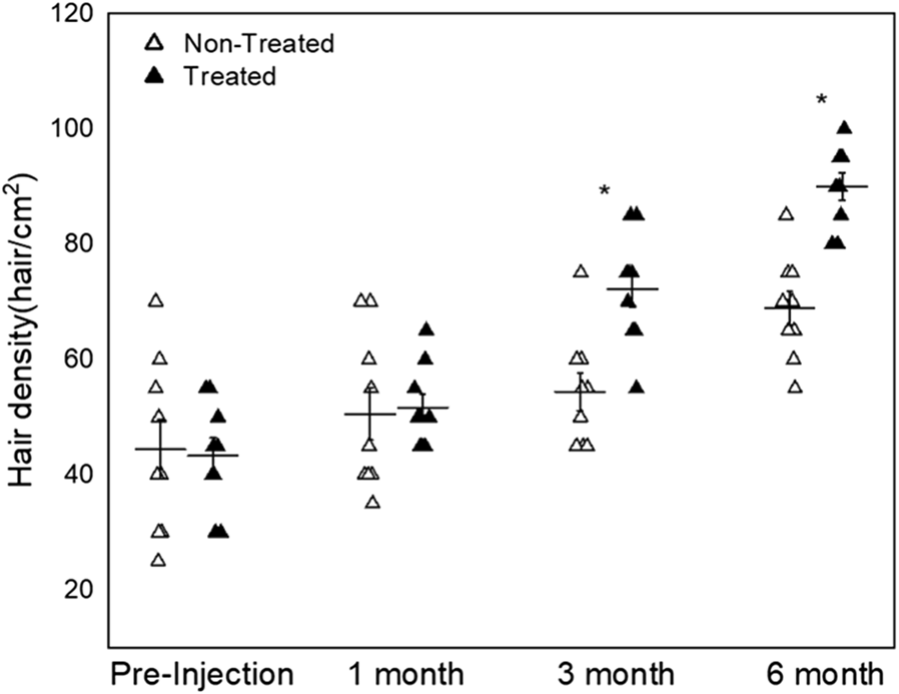
Analysis of hair density change before and after the treatment of SVF. The difference of median value between treated and non-treated side is significantly difference at 3 and 6 months (*Wilcoxon signed-rank test, *P* < 0.05, *n* = 9)

Both groups, the scalp images were taken and the number of hair/cm^2^ was counted randomly in the transplanted area on each visit. In one patient, based on AGA condition, we randomly treated a half with SVF, while the other part was not done. Mean and median density of hair on the pre-injection visit in the non-treated site were 44.44 ± 5.09 vs. 43.33 ± 3.11 in the treated site (Fig. 1). On 6 months after treatment, the number of hair density was in the non-treated site 68.88 ± 2.97 versus 90 ± 2.35 in the treated site. Overall, the density was increased in the treated site by 48.11% as compared to the non-treated site density of 35.48%. Hair density of the treated side was highly improved after 3- (*P* = 0.01, *n* = 9) and 6-month pre-injection (*P* = 0.009, *n* = 9) (Fig. 1).

**Fig. 2:**
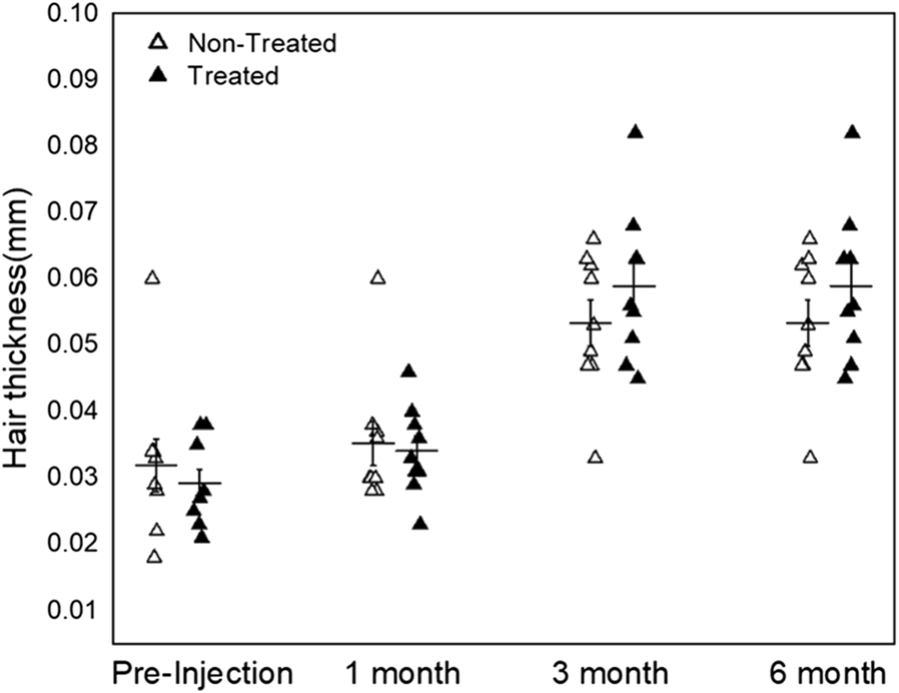
Changes of hair thickness before and after the treatment of SVF. The difference of median value between treated and non-treated side was increased at 3 and 6 months. However, there was no statistical significance (Wilcoxon signed-rank test, *n* = 9)

**Fig. 3:**
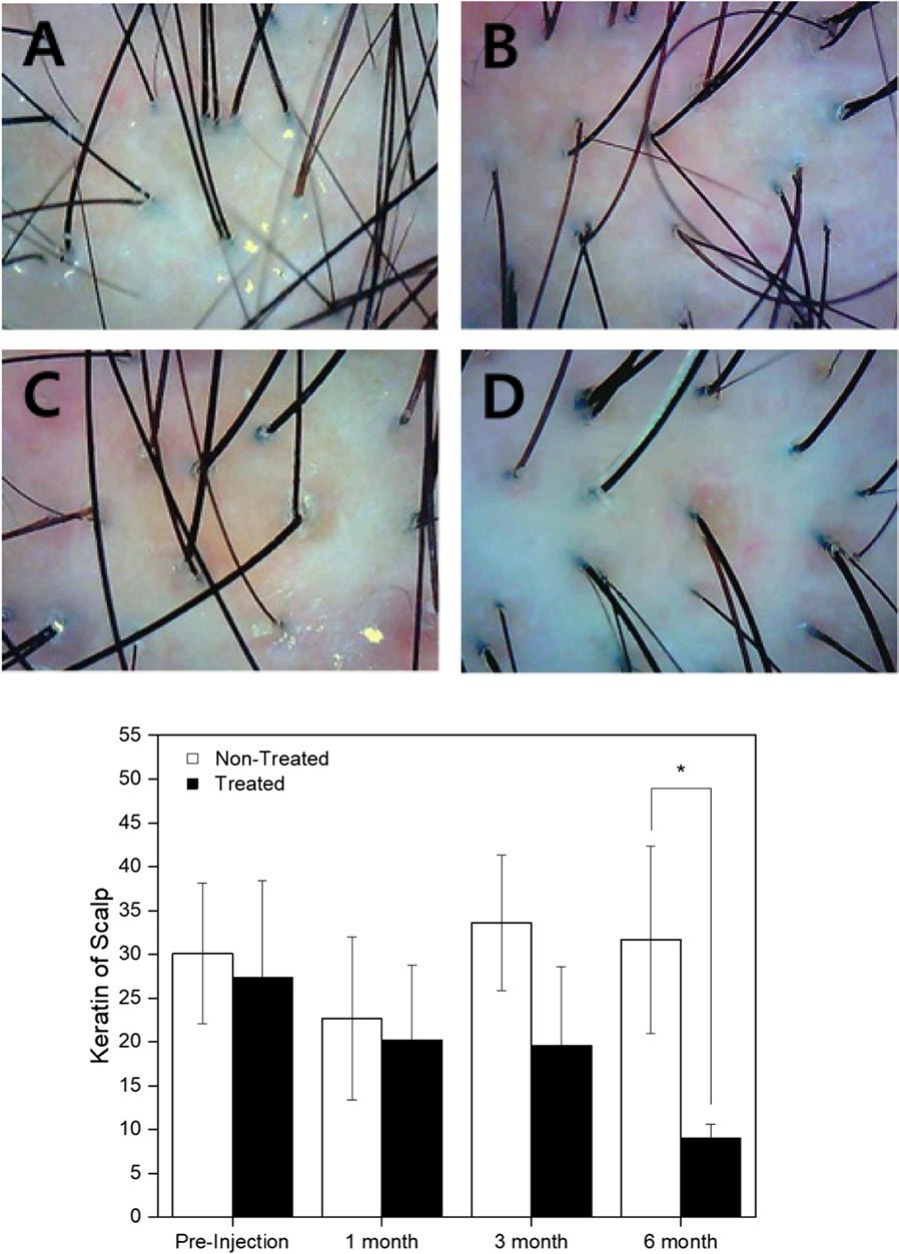
Representative image of keratin on scalp pre-injection and 6 months after SVF treatment. The median value between treatment and non-treatment side was significantly different at 6 month (Wilcoxon signed-rank test, *P < 0.05). 51-year-old men (**A**, **B**) and 43-year-old woman (**C**, **D**) decreased the score of keratin on scalp after 6 months post-injection

**Fig. 4:**
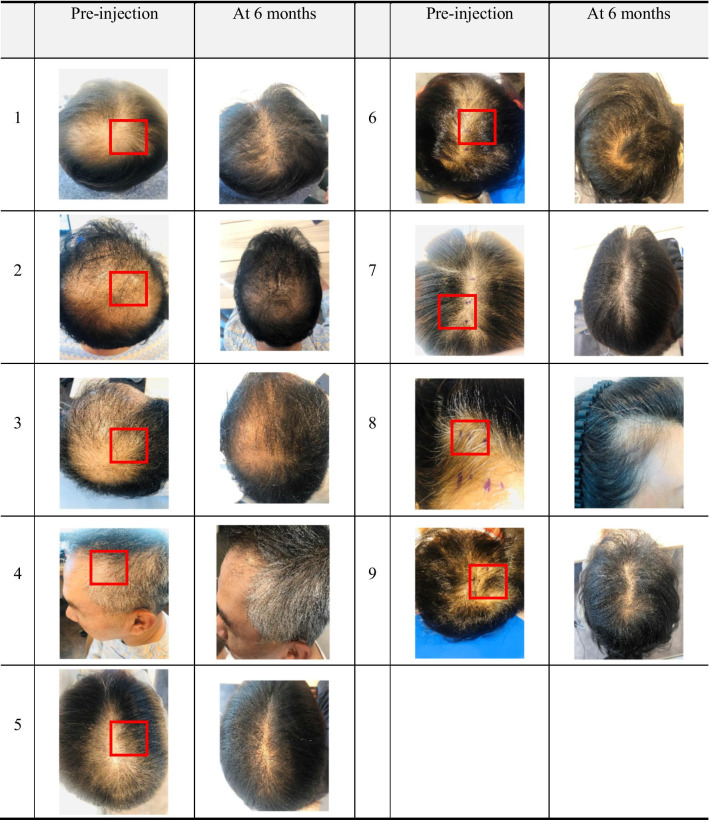
Representative photographs of the AGA improvement after SVF treatment. Baseline (pre-injection) versus 6 months (post-injection) global photographs after treatment

Hair thickness improvement was observed after 3 and 6 months post-injection, but there was no statistical significance (Fig. 2). The mean and median thickness of hair on the pre-injection visit were 0.032 ± 0.053 mm in the designated non-treated site compared to 0.029 ± 0.003 mm in the designated treated site. On the 6 months post-injection visit, hair thickness was 0.053 ± 0.003 mm in the non-treated site compared to 0.058 ± 0.003 mm in the treated site. Any overall significant change in the hair thickness, scalp status, sensitivity, sebum, hair pore status, cuticle status, or any other parameters was not shown in the treated area at whole 6-month follow-up except hair density and keratin of scalp (Fig. 3). Although the patients do not achieve any improved scores in their hair status, significant improvement in the score for the keratin of the hair epidermis was seen in the treated side as compared to the non-treated side (*P* = 0.032). After 6 months, most of the patients showed improvement in the hair status and patient satisfaction scores (data not shown). Also, the pull test was done in both sides of the patients after 6 months, and there is no significance compared to non-treated group (data not shown). Representative photographs and macrophotographs of a patient after 6 months are shown in Fig. 4.

## Discussion

This study investigated the potential role on the SVF on AGA. Similar to MSCs, SVF is available in large quantities from the abdomen or thigh in a relatively less invasive liposuction, which is considered as a useful tool for the cell-based treatment, just like bone marrow-derived stem cells. SVF consists of ASCs, endothelial cells, pericytes, macrophages and other immune-related cells which secrets neovascular factors responding to ischemia or stimulates growth factor. Due to these characteristics of SVF, stem cells using minimal manipulation have been a very active topic in many studies and so, the concerned clinical studies on SVF have been highly popular [14].

Recently, improvement of AGA treatment with SVF has been used as an effective method in the various translational researches. Using cultured or uncultured SVF [17], regeneration, immune control and angiogenesis promotion and its corresponding utility have been spread in the clinics in the easiest and most promising way [19, 20]. Recently, there are studies using PRP or autologous MSCs [10, 11] for AGA treatment, and it has been reported that AGA improvement using SVF is more effective and discovery of cell secretion for the cell death/necroptosis regarding hair follicle will be a breakthrough to AGA [21]. But it is necessary that careful design of microenvironment to activate SVF, development of cell transplantation protocols to maximize the capabilities of SVF while preventing unexpected cell behavior and the proper selection of target diseases will be also a critical factor to lead successful clinical applications.

In this study, the therapeutic role of SVF was assessed by AGA-related criteria such as hair density, thickness, and other status of scalp. The median hair density on the treated side was significantly increased compared to the non-treated side (*P* = 0.009 and 0.032, respectively). However, although increase in the thickness was observed at 3 and 6 months post-injection, there was no statistical significance (*P* = 0.142 and 0.155, respectively). This result is thought to be related to the thickness of newly generated hair and so it is necessary to extend the observation period. In relation to the status of scalp, functionless hair follicles full of hyperkeratotic plugs [22], assumed incapable of making new hair grow, showed more significant improvement in the score for the keratin of the scalp in the treated side as compared to the non-treated side (*P* = 0.032).

So far, one of representative AGA treatments is to use anti-androgen drugs which suppresses male hormone and the other one is to use minoxidil. In addition, new anti-androgen drugs and medical devices are currently being developed and hair follicle regeneration research using some follicle cells is ongoing. Among these treatments, it is known that it is difficult to do long-term use for anti-androgen drugs, due to the side effects of inhibiting male hormone. In case of minoxidil, hair loss inhibition is not appeared to all patients and there is dissatisfaction with sense of use. Other technologies such as hair follicle transplantation and medical devices have been developed, but there are some limitations such as cost burden and somewhat weak effectiveness [22–26].

The results of this study show that approx. 48% of the hair density has been improved after the transplantation of autologous SVF. The improved effect of the hair loss using autologous SVF can be a good treatment model for men as well as women. Furthermore, the improvement of hair loss using SVF is considered to have a good, expected effect on AGA when used in combination with existing biological treatment methods such as follicle stem cells therapy [27]. Though previous study for animal models on hair growth has been published, there are not so many cases in clinical trials to improve or inhibit AGA using SVF [28]. In the recent examples when minoxidil, an AGA treatment agent is used in combination with autologous SVF transplantation, and it has been reported that hair growth is improved, which is known as paracrine effect by the migration of transplanted SVF and the secretion of various growth factors [29]. As it is known, SVF secrets various cytokines related to immunosuppressive action or anti-inflammation by interaction of various cells [30].

In the application of clinical study using SVF, the type, structure, and surroundings of damaged tissue/organ have an important influence on the transplanted SVF [31, 32]. Efficacy on SVF-based therapy in AGA depends on several variables such as optimal cell number, phenotype, maturity formulation and transplantation method. Like other stem cells, the fate of transplanted SVF is determined by various microenvironments such as apoptosis, extracellular material decay, bleeding, inflammation, hypoxic environment, cytokine, tissue damage, mechanical strength, and other factors. Furthermore, it is necessary to study the role of numerous cells, the preparation process, intercellular interactions, extracellular substrates, growth factors and biomaterials, "on/off" signaling pathways, and the microcellular environment acting at each stage of tissue/organogenesis [33].

This study did not complete blind process for subjects and has a limitation for a small number of subjects to recruit. Despite these limitations, this study presented fundamental improvement of AGA using autologous SVF and in the future, progressive study is required to be carried out by improving research design.

This strategy can suggest not only a treatment itself for AGA but also be helpful to develop regenerative medicine applications successfully. But, to overcome the limitations in this study, AGA treatments with various causes and complex mechanism, therapeutic agent development for AGA is considered to be essential. As the underlying cause of AGA is prevented by SVF, if the long-term safety and efficacy are secured, compared to conventional treatment method, it is expected to provide an effective method to cure AGA in the future.

## Conclusions

This study using autologous SVF can be regarded as a cell therapy method to differentiate from existing AGA treatments. This study proved to improve and maintain hair density for more than 6 months through single injection of SVF and verified the improvement of some criteria on the effect of AGA inhibition. In the future, additional studies on more suitable transplant methods using SVF and establishment of clinical treatment protocols for improving effects on AGA will be also required to be done.

## Data Availability

All concerned data or analysis included in this study are available in the article.

## Abbreviations

ADSCs: Adipose-derived stem cells
MSCs: Mesenchymal stem cells
AGA: Androgenetic alopecia
SVF: Stromal Vascular fraction
PRP: Platelet-rich plasma
